# Emerging Links between Microbiome Composition and Skin Immunology in Diaper Dermatitis: A Narrative Review

**DOI:** 10.3390/children9010112

**Published:** 2022-01-15

**Authors:** Tjaša Hertiš Petek, Maya Petek, Tadej Petek, Nataša Marčun Varda

**Affiliations:** 1 Department of Pediatrics, University Medical Center Maribor, 2000 Maribor, Slovenia; tadej.petek@ukc-mb.si (T.P.); natasa.marcunvarda@ukc-mb.si (N.M.V.); 2 Faculty of Medicine, University of Maribor, 2000 Maribor, Slovenia; maya.petek@um.si

**Keywords:** diaper dermatitis, immunology, inflammation, microbiome, nappy rash, pediatrics, pH, probiotics, skin

## Abstract

Diaper dermatitis is a common type of irritant contact dermatitis occurring in infants and toddlers. Its occurrence is triggered by an unfavorable environment under the diaper, damage to skin integrity by fecal enzyme degradation, overhydration and disruption of the lipid bilayer structure facilitating the entry of irritants and microorganisms. In diaper dermatitis development, the central proinflammatory cytokines are IL-1α, IL-8 and TNF-α. The initial release of IL-1α and TNF-α starts a further cascade of pro-inflammatory chemo- and cytokines, resulting in inflammation and erythema of the skin. A recently recognized factor in diaper dermatitis is the composition of the skin microbiome; common pathogenic strains *Candida albicans* and *Staphylococcus aureus* are associated with skin irritation. The resulting impaired microbiome composition produces a local inflammatory response and may thus worsen the initial dermatitis clinical presentation and subsequent healing. Introduction of probiotics is an attractive treatment for microbiome modulation, which has shown success in other skin conditions in adults and children. Probiotics are thought to work as a protective shield against irritants, maintain low skin pH, secrete beneficial metabolites, and block pathogen invasion. There is preliminary evidence that certain probiotics given orally or topically could be used as a gentle intervention in diaper dermatitis.

## 1. Introduction

Diaper dermatitis (DD), also known as diaper/napkin/nappy rash, encompasses various infant dermatoses occurring in the perineal and perianal area [1]. These eruptions may represent exacerbations of diffuse skin diseases, such as seborrheic dermatitis or atopic dermatitis (AD), or skin conditions that coincidently manifest in the diaper covered area [2]. This review uses the stricter definition, where DD is defined as a type of irritant contact dermatitis (ICD), occurring mostly in reaction to prolonged contact with urine, feces, or retained soaps and detergents (falling under the ICD10 diaper dermatitis L22 diagnosis), resulting in an acute inflammatory skin process [3].

As one of the most prevalent skin conditions affecting babies, DD presents a problem to many parents after the birth of their child [4]. It usually occurs in the first two years of life, especially between 9 and 12 months of age [5,6]. A recent study estimated the prevalence of DD in children under the age of 2 at around 36%, which decreased significantly with increasing age [7]. DD typically affects the lower abdomen, thighs, and the entire area under the diaper, including intertriginous folds. DD initially presents with desiccation of the skin, followed by development of erythematous maceration and oedema [3].

Few studies so far have addressed the specific mechanism of skin inflammation in diaper dermatitis [8,9,10,11]. While research on the skin microbiome in the diaper microenvironment has already been reviewed [12], evidence is lacking on the interplay between inflammatory processes and the corresponding microbiome in the diaper area. Here, we review the literature on the skin microbiome composition and inflammatory processes in children with diaper dermatitis, focusing on whether reversal of inflammation via alteration of the diaper skin microbiome might be achieved.

## 2. Methods and Materials

A PubMed, MEDLINE, CINAHL, Cochrane Library and Google Scholar search was conducted independently by two investigators (T.H.P., T.P.) for search terms “diaper dermatitis”, “napkin rash”, ”nappy rash”, “eczema”, “atopic dermatitis”, “irritant contact dermatitis”, “incontinence associated dermatitis”, “skin”, “probiotics”, “pediatrics”, “inflammation”, “inflammatory process”, “pH”, “microbiota”, and “microbiome”. A manual search of relevant reference lists was also performed.

For this narrative critical review, original research and review articles in English and Slovene languages reporting results in children and adults were included. There was no limit on the publication date, although we aimed to include studies within the last 20 years. Any discrepancies regarding the inclusion of a study were discussed with another investigator (M.P.) and resolved.

The included studies were further assessed for the quality of the design and of the results presented. No standardized study assessment tools were used. If applicable, the potential bias of an industry-sponsored study was evaluated and discussed in the manuscript text. Both positive and negative studies were included. Due to the small number of pediatric studies on inflammation, dermal microbiome and probiotics use in diaper dermatitis, applicable studies on other types of dermatitis in children and adults were also included in the second stage of literature acquisition.

## 3. Results

### 3.1. Early Fetal and Postnatal Microbiome

Adult skin is mainly colonized by four different phyla with quite stable dominant genera: Actinobacteria (most dominant reported genera: *Propionibacterium* and *Corynebacterium*), Firmicutes (most represented by *Lactobacillus*, *Streptococcus* and *Staphylococcus*), Proteobacteria and Bacteroidetes, respectively [13].

Dermal bacterial colonization is generally thought to begin at birth and continues forming throughout the first years of life and into adulthood [14,15]. The mode of fetal delivery is recognized as the major determinant of the newborn′s cutaneous microbiome composition [15]. Recently, some authors proposed that maternal microbiota is selectively transported to the placenta to colonize the fetus already before birth. This was supported by reports of bacterial DNA in the placenta and amnion [16]. The presence of oral and meconium microbiota has been reported at the time of Caesarean delivery, supposedly originating from the placenta [17]. It appears this early lack of sterility may be protective. Non-sterile meconium-stained amniotic fluid is present in 5% to 20% of deliveries and was reported to reduce the risk of developing dermatitis and decreased skin-eruption-related hospitalizations throughout childhood and adolescence [18].

Immediately after birth, infant skin bacterial communities have been reported to be similar across body sites [19]. As soon as two days after delivery, bacterial communities begin to diverge into distinct functional communities at different skin sites, which are similar to those found in adults [20]. The buttock skin bacteria soon form a separate microbiome environment, due to less likely competition from other skin sites to the diaper covered area and proximity to the gastrointestinal tract [21]. Commonly isolated bacteria from the diaper area of neonates less than a week old are species of *Bifidobacteria* and *Bacteroides*, followed by *Enterobacteria, Eubacteria, Lactobacilli,* amongst others [12,22].

Site-specific evolution of different bacterial communities appears to happen within the first three months of life [21]. Six weeks after delivery, the skin microbiota of mother and infant are more similar than their microbiota at other body sites, like the gut and oropharynx, which diverge more rapidly [23]. Over the first year of life the similarity between mother and infant microbiota further decreases as successful invasion of the environmental strains becomes more important [24]. In time, the infant’s own gastrointestinal flora becomes an important contributor to the microbiota under the diaper, mainly colonized with *Clostridium* species (spp.) and other gut-derived bacteria, and also significant amounts of *Bacteroides* spp. [12].

The gastrointestinal microbiota depends on various factors, especially the child’s diet: breastfed, bottle-fed or already weaned [25,26,27]. Numerous indicators suggest important benefits of breastfeeding for child’s health during infancy and later in life [28]. Because of the oligosaccharide content, breastfeeding stimulates intestinal proliferation of anaerobic microorganisms such as species of *Bifidobacteria (B. breves*, *B. infantis*, *B. pseudocatenulatum)*, *Lactobacilli* and *Bacteroides*. Bottle-feeding develops a mixed bacterial flora in the infant intestine, with a reduction of *Bifidobacteria* spp. and a greater presence of *Bacteroides* spp., *Clostridia* spp. and *Staphylococci* spp.. When breast-feeding is supplemented with bottle-feeding, the profile of intestinal microflora is similar to formula-fed infants [25,26,27]. Weaning is the main milestone when the intestinal flora becomes similar to that of an adult, with an increase in *Bacteroides* spp. and anaerobic gram-positive bacteria, such as *Peptococci* spp., *Peptostreptococci* spp., *Veillonella* spp. and *Staphylococci* spp. [27].

Early in life, the microbiome is highly plastic. It undergoes dynamic changes until the age of 3 to 4 years, when it stabilizes and becomes more adult-like [29]. Because of the shifting nature of microbiomes in preschool children, we believe probiotics might be especially beneficial in treating babies with DD.

### 3.2. The Microbiome in Diaper Dermatitis

The buttock area is unique in its microbiota composition with an early colonization with aerobic bacteria (*Staphylococcus* spp., *Streptococcus* spp. and *Enterococcus* spp.) and other transitional taxa such as *Prevotella* spp., *Veillonella* spp. and *Clostridion* spp.. In children with DD, the affected skin is highly susceptible to microbial infections, in particular to intestinal microbial residues [30]. In addition, both secondary bacterial and *Candida* infections can complicate dermatitis [3]. In DD specifically, colonization with *Finegoldia* [31] has been proposed as an important contributing factor because of its higher-than-expected abundance on the buttock skin area, but the mechanism is still unclear [21]. An increased presence of *Enterococci*, which are normal skin and gut bacteria with a pathogenic potential, has also been shown [32].

These results show that the composition of the normal skin microbiota in diaper dermatitis is altered. Bacterial diversity in DD patients is higher compared with healthy controls [30]. This contrasts the generally accepted notion of a beneficial effect of greater bacterial diversity, as seen in AD [13].

When it comes to skin flora, the DD affected area showed a paucity of beneficial strains like *Staphylococcus epidermidis*, *Bifidobacterium longum*, *Clostridium butyricum* and *Lactobacillus ruminis* [30]. Although the colonization with *Staphylococcus* spp. decreases with increasing DD severity, the more severe DD the predominant *Staphylococcus* spp. is *Staphylococcus aureus*, potentially implicating *S. aureus* as a DD etiological agent. In contrast, the demonstrated community percentages of fecal coliforms increase with DD severity [33]. The most often isolated pathogenic strains are *Candida albicans* and *S. aureus*. It is reasonable to believe that they play a predominant role in DD [12], but further research on the impact of microbiome composition on DD is certainly needed.

### 3.3. Inflammation in Diaper Dermatitis

**Figure 1:**
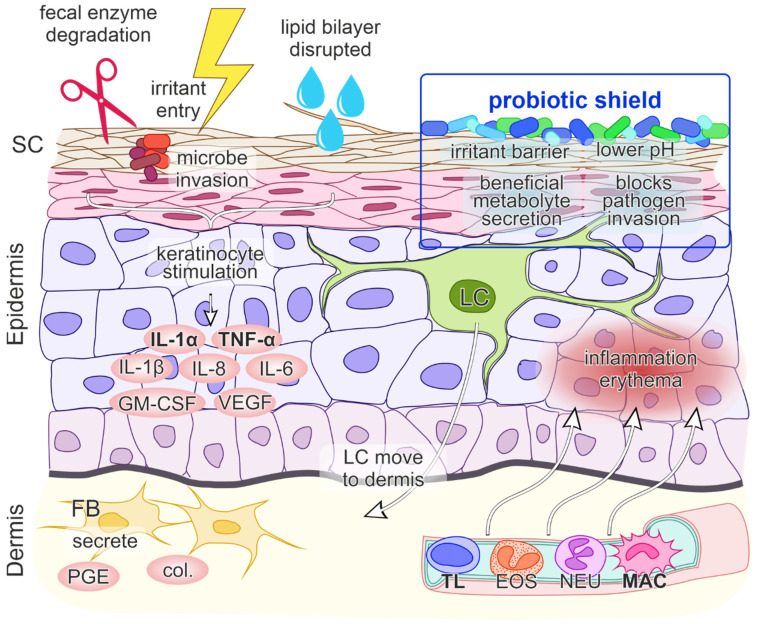
A model of contact irritant diaper dermatitis. The first step involves the penetration of irritants through the skin which stimulates keratinocytes (KC) to release proinflammatory mediators interleukin 1 alpha (IL-1α) and tumor necrosis factor alpha (TNF-α). The entry of irritants and microorganisms is facilitated by damage of stratum corneum (SC) integrity by fecal enzyme degradation, overhydration and disruption of the lipid bilayer structure. The initial release of IL-1α and TNF-α promotes further production of cytokines and chemokines IL-1β, granulocyte macrophage-colony stimulating factor (GM-CSF), IL-6, IL-8, vascular endothelial growth factor (VEGF), migration of Langerhans cells (LC) to the dermis, production of collagenases (col.) and prostaglandin E (PGE) by fibroblasts (FB), vasodilatation of the blood vessels, upregulation of adhesion molecules on endothelial cells and the transmigration of inflammatory cells (TL—T lymphocyte, EOS—eosinophil, NEU—neutrophil, MAC—macrophage) to the epidermis. The net effect is inflammation and erythema of the skin. Probiotics present a protective shield against irritants, maintain a lower pH, secrete beneficial metabolites and block pathogen invasion.

Skin inflammation is generally triggered by external factors such as allergen intake, contact with microbes or with irritants, UV radiation and by other, less well-defined stimuli [34]. In DD skin irritation is affected by adverse fecal enzymes, friction and subsequent skin maceration, high pH, the presence of urine and prolonged skin contact with feces [6]. Inadequate skin care, certain microbial invasion, antibiotic use and a lack of individual nutrients also play a role (Figure 1) [3]. The resulting inflammatory process involves a complex interplay of events between skin cells, immune cells, inflammatory cytokines, and chemokines [35].

Interleukin 1 alpha (IL-1α) is a major driver of inflammation in DD, as infants with DD, heat rash and erythema show a significant increase in IL-1α levels compared to healthy infants. There is a strong positive correlation between interleukin 1 receptor antagonist (IL-1RA) levels and DD severity. In the DD area authors also observed a significant increase in interleukin-8 (IL-8) levels in comparison to healthy skin sites [11]. Examination of an in-vitro skin model confirmed different formulae for alleviation of DD, noticing that chemical irritation causes a high release of IL-1α, impairs tissue viability as well as skin barrier integrity [10].

The importance of IL-1α was further demonstrated in the first month post-partum: levels of IL-1α and skin hydration were significantly increased at day 28, while the pH of the skin decreased regardless of the skin care regimen. IL-1α levels were significantly higher in the skin of infants wearing diapers compared to those without, while skin cleansing with care cloths or water showed no differences in microbiological colonization. Authors proposed that an increase of IL-1α might reflect postnatal skin maturation [8]. In a similar study from 2014, authors described higher skin pH, increased skin hydration and increased IL-1α concentrations in infants who used diapers compared to those without [9].

### 3.4. Role of Skin pH

Across body sites pH values vary from pH 4.0 to 7.0. Around half reports describe pH values below 5.0, which contrasts with the general assumption of a healthy skin pH being between 5.0 to 6.0. Low pH values below 5.0 are associated with favorable barrier function, moisturization and scaling. A proposed mechanism for low pH protective effect is that an acidic skin pH between 4.0–4.5 keeps the resident bacterial flora attached to the skin, whereas an alkaline pH between 8.0–9.0 promotes dispersal from the skin [36]. Maintenance of the acidic layer of the epidermis is also of major importance as it maintains a protective system of the skin and creates an unfavorable environment for colonization by pathogenic microorganisms [37]. Ammonia-induced alkalinization activates fecal enzymes such as lipase and trypsin, leading to irritation and disruption of the skin barrier. However, the skin in some cases of infants has already been reported to have an elevated baseline pH of 6.6 [38], suggesting a possible predisposition for DD development.

A normal pH value in the buttock area is around 5.5 [39] and any increase in skin pH in the area under the diaper promotes the growth of pathogenic microorganisms, including *C. albicans* and *S. aureus*, which seem to play a predominant role in DD [12,40,41]. The inflammatory process of the skin in the buttock area is related to specific bacterial strains that promote inflammation. For example, a characteristic of *S. aureus* infection is the formation of a neutrophilic abscess. This infection is generally characterized by inflammatory cytokines interleukin-1 beta (IL-1β), tumor necrotizing factor alpha (TNF-α), interleukin-6 (IL-6), chemokine (CX-C motif) ligand 2 (CXCL2), and IL-8 (also known as chemokine (CX-C motif) ligand 8-CXCL8) [40]. In *C. albicans* infection, the cytokines IL-6, TNF-α, interleukin-12 (IL-12), interferon-γ (IFN-γ) and interleukin-17 (IL-17) appear in the initial stage of skin infection. On the other hand, anti-inflammatory cytokines interleukin-13 (IL-13), interleukin-4 (IL-4), interleukin-10 (IL-10) and transforming growth factor-β (TGF-β) appear on the fourth day after infection and ameliorate the exacerbation of inflammation and participate in the healing of lesions [41].

Furthermore, the humid environment leads to overhydration of the stratum corneum (SC), causing disruption of the lipid bilayer structure. When the SC integrity is damaged, irritants and microorganisms can easily penetrate and reach the epidermis and Langerhans cells. Penetrants/irritants interact with keratinocytes, stimulating the release of cytokines, which then act on dermal vasculature, resulting in inflammation (Figure 1) [42,43,44].

Although skin pH, microbiome, and inflammation have been studied separately, further work is needed to elucidate connections between these factors. So far, an increase in pH is thought to increase the release of inflammatory biomarkers, specifically interleukins, such as IL-1α. It has been proposed that IL-1α and TNF-α could serve as biomarkers of skin damage caused by urinary incontinence [45]. The question whether the increase of the skin pH by itself causes the inflammation or only provides suitable conditions for pathogenic colonization that afterwards causes the inflammation deserves to be further evaluated.

### 3.5. Inflammatory Signaling

At a molecular level, when DD results from a contact etiology, irritant penetration through the skin induces endogenous “danger” signals, which cause direct damage to keratinocytes and the release of cytokines and chemokines IL-1α, IL-1β and TNF-α. These cytokines unsurprisingly play a central role in DD immune system activation, as they can trigger inflammation on their own. Keratinocytes additionally secrete the granulocyte-macrophage colony-stimulating factor (GM-CSF), IL-6 and IL-8, where IL-8 is a potent chemokine for lymphocytes and IL-6 influences the process of maturation of keratinocytes. The initial signal release is followed by the migration of Langerhans cells to the dermis, the production of collagenases and prostaglandin E by fibroblasts and the upregulation of intercellular adhesion molecule 1 (ICAM-1) and vascular cell adhesion molecule 1 (VCAM-1) on keratinocytes, fibroblasts and endothelial cells. Subsequently, blood vessels dilate and passage of inflammatory cells into the epidermis ensues, leading to inflammation and erythema (Figure 1) [35,46].

Less well characterized cytokines that are involved in ICD are CCL (chemokine (C-C motif) ligand) 20, CCL27, IL-10, IL-12 and IL-18 [35]. Of these, IL-2 is the most important growth factor for T lymphocytes while IL-10 inhibits cytokine synthesis and thus the immune response [46]. A synergistic effect of cytokines IL-1 and TNF-α has been described, leading to further activation and release of secondary chemo- and cytokines such as IL-2, IL-6, GM-CSF, IFN-γ, vascular endothelial growth factor (VEGF), IL-8, CCL2, CCL5 and CCL20, which further stimulate the expression of the cellular adhesion molecules [43].

The cytokines that are primarily upregulated following irritant exposure are IL-1α and TNF-α [35,43,46]. Although the precise cytokines/chemokines activation cascade in DD is still unclear, IL-8 has also been specifically implicated as a strong contributor in DD [42].

The importance of IL-1α and TNF-α in ICD is supported by genetic findings, showing that individuals with *TNFA*-308A alleles have an increased risk of ICD while those with *TNFA*-238A polymorphisms have a reduced risk [47]. The single nucleotide polymorphism (SNP) of *IL1A*-889T was also associated with less likely development of ICD [48]. SNPs involved in the development of ICD in health professionals were within the MHC Class I (*OR2B3, TRIM31, TRIM10, TRIM40* and *IER3*), Class II (*HLA-DPA1, HLA-DPB1*) and Class III (*C2*) genes and were associated with skin response to tested irritants in different genetic models. Linkage disequilibrium patterns and functional annotations identified two SNPs in the *TRIM40* (rs1573298) and *HLA-DPB1* (rs9277554) genes, with a potential impact on gene regulation [49]. These results suggest that polymorphisms of individual nucleotides, associated with skin inflammation and homeostasis, can affect responses to irritants and at least partially explain individual differences in the development of contact dermatitis. IL-1α, IL-8 and TNF-α seem to be the most important cytokines in DD [8,9,10,11,42]. Inflammatory processes depend not only on the trigger but also on genetic predispositions [34,35]. However, the exact cytokine profile in DD is still unknown and likely varies between patients, the microbiome composition and the irritant nature.

### 3.6. Probiotics as a “Protective Shield” against Skin Inflammation

Probiotics are live microorganisms which, when administered in adequate amounts, confer a health benefit on the host [50]. Recently the understanding of the microbiome’s role in skin disease has grown, confirming that the immune system can be modulated through the introduction of specific probiotics [51].

Probiotics exert health effects on the skin directly through cutaneous formulations or indirectly through dietary supplementary formulations and intestinal microflora improvement [52]. Certain probiotics can modulate the cutaneous microflora, the lipid barrier, and the skin immune system, leading to the maintenance of skin homeostasis [53].

Beneficial dermal effects of probiotics have been shown via oral consummation through acting on the intestine with changes in systemic immune responses and thus immunomodulation of the skin, inhibition of allergen-induced tumors via changes in systemic immune responses and inhibition of harmful intestinal microflora. Probiotics can also act as antioxidant agents. Probiotics may be applied directly on the skin which then compete with harmful skin microflora, secrete useful metabolites, reduce pH and act as a barrier to harmful foreign environmental factors that are in contact with the skin (Figure 1) [52].

As the interest in the use of probiotics in DD is very recent, there is currently only very limited data available on their use as a DD prevention or treatment option. An older preliminary report from 1998 describes a trial of infant formula supplemented with *Bifidobacterium lactis* and *Streptococcus thermophilus*, which showed a small decrease in DD incidence compared to non-supplemented formula during the observation period [54]. Examining probiotics as a DD treatment, a single 2021 article reports on a market research study that followed an at-home use of oral activated *Bifidobacterium infantis* EVC001 in infants with DD. Parent-reported changes in DD severity, symptomatic relief of colic and sleep behaviors were collected via an online survey and showed promising outcomes: 72% (*n* = 448) of the participants whose infants had ever experienced diaper rash reported improvements, and 57% of those reported complete resolution of diaper rash [55]. Although the consumer feedback was very positive, this type of research presents substantial methodological limitations; there is little opportunity to monitor confounding factors and possible bias incentives. Further, more rigorous scientific research on use of probiotics in DD is desired.

In children the evidence for probiotics use exists mainly for AD [27], for which a recent systematic review on use of probiotics in AD concluded that certain probiotic preparations were efficient in reducing risk of developing AD when administered to pregnant women, infants, or both [56]. A number of probiotic preparations showed an effect: a mix of *Lactobacillus paracasei* ST11 and *Bifidobacterium longum* BL999; the *L. paracasei* ssp. *paracasei* strain F19; *L. rhamnosus* GG with *B. animalis* ssp. *lactis* BB-12 [56]. An older review study concluded that *L. rhamnosus* GG and *B. lactis* BB-12 showed an effect when adjunct to extensively hydrolyzed formula in treating infants with mild AD and cow’s milk allergy, and the combination of *L. rhamnosus* 19070-2 and *L. reuteri* DSM 122460 was effective in patients with moderate to severe AD [27].

Although the skin barrier is altered in both AD and DD, the pathogenic mechanism differs between the two types of dermatitis. In searching for evidence of probiotics use in childhood dermatitis, we searched for studies in ICD (which would be the most relevant), but no results were found. Instead, the closest relevant results were found for eczema in general, not specifying whether atopic, contact, dyshidrotic, seborrheic etc. In eczema, the evidence is mixed: *Lactobacillus* and *Bifidobacterium* strains showed a protective effect, although *Bifidobacterium* was not tested unilaterally [57]. In contrast, a recent systematic review concluded that currently available probiotic strains probably make little or no difference in improving patient-rated eczema symptoms. Results show significant, unexplainable heterogeneity between individual trial results. The authors suggest that in the future, researchers should also consider studying subgroups of patients (e.g., patients with atopy or food allergies, adults) and standardize the doses/concentrations of probiotics given [58]. In summary, the heterogeneity of terms and definitions regarding eczema is the major limitation of studies that reported effect of probiotics on the skin, limiting the possibility of generalization [57]. The current consensus recommendation of the International Eczema Council (IEC) is to use of the prefix “atopic” (i.e., AD or AE) in all publications, presentations and discussions about the disorder [59]. We expect that the quality of the available evidence will improve with more precise reporting of the treated skin pathology.

Finally, due to limited reports on probiotic interventions for dermatitis in children, we expanded our search to include relevant studies in adult populations. Positive results for probiotic interventions in adults were reported for acne vulgaris, psoriasis, diabetes skin ulcers, acute and chronic wounds, skin cancers, burns, seborrheic dermatitis, photoaging and allergic contact dermatitis [51,60]. Most studies investigated oral probiotic interventions, and of those utilizing topical probiotics, few included skin commensals [51]. Some authors also present positive clinical findings on gut-skin axis interventions with oral probiotics [61,62]. Although we searched extensively for the use of probiotics in ICD or incontinence associated dermatitis in adults, which would be most applicable for DD in children, there were no studies reported.

However, there are some adult studies that tested the effect of probiotics on skin barrier function. In one trial, participants were consuming *L. brevis* SBC8803 oral supplements for 3 months, which resulted in decreased transepidermal water loss (TEWL) and increased corneal hydration [63]. In a placebo-controlled human study, bacterial supplementation had a positive effect on skin barrier function. When participants were taking *L. paracasei* NCC2461 supplements for 8 weeks, they developed decreased skin sensitivity and TEWL. Circulating TGF-β levels, a cytokine that is beneficial for a barrier integrity, were increased after the use of probiotics [64].

Even though studies on the skin barrier function examined the effects of probiotics on undiapered adult skin, the results might be relevant to DD as the formation of ICD in DD is closely related to the maintenance of skin barrier integrity. Still, DD specific trials are certainly needed because the generalizability is limited by factors including different study populations (adult vs. infant) and skin environment effects (diaper coverage).

## 4. Conclusions and Outlook

Although the immediate cause of DD is contact with environmental irritants, the individual predispositions to its development and severity are less clear. The microbiome composition seems to be an important factor. Evidence for microbiota modulation with probiotics in children exists mainly for atopic dermatitis (AD), where some studies reported positive results [27].

The exact pathophysiological mechanism of probiotic immune system modulatory effects and counteraction of inflammation processes in DD is yet to be understood. IL-1α, TNFα and IL-8 seem to play a central role in driving DD inflammation, but exact involvement of other cytokines and chemokines is still to be uncovered. Furthermore, the interplay of inflammatory pathway activation and the microbiome require further investigation.

When it comes to diaper rash, Zheng and colleagues recently reported that altered skin microflora indicates imbalance and dynamism of microbiome in diaper rash. They were one of the first to suggest that the use of probiotics could be potentially a successful strategy for the prevention and treatment of diaper rash [30]. The use of probiotics in DD is a promising research direction, as probiotic intervention clinical trials have yielded positive results in various skin conditions in adults and children.

We believe that probiotics are a promising, gentle intervention for children with DD, and their use warrants further exploration. Such trials should be supported by additional research into greater understanding of the correlation between inflammation and microbiome in DD.

## Data Availability

All the data are available within the manuscript.
